# Doctor shopping among chronic noncancer pain patients treated with opioids in the province of Quebec (Canada): incidence, risk factors, and association with the occurrence of opioid overdoses

**DOI:** 10.1097/PR9.0000000000000955

**Published:** 2021-09-16

**Authors:** Jean-Luc Kaboré, M. Gabrielle Pagé, Lise Dassieu, Éric Tremblay, Mike Benigeri, Denis A. Roy, Anaïs Lacasse, Manon Choinière

**Affiliations:** a Department of Pharmacology and Physiology, Faculty of Medicine, Université de Montréal, Montreal, QC, Canada; b Research Centre of the Centre hospitalier de l’Université de Montréal (CRCHUM), Montreal, QC, Canada; c Institut national d'excellence en santé et services sociaux (INESSS), Montreal, QC, Canada; d Department of Anesthesiology and Pain Medicine, Faculty of Medicine, Université de Montréal, Montreal, QC, Canada; e Department of Psychology, Faculty of Arts and Science, Université de Montréal, Montreal, QC, Canada; f Department of Health Sciences, Université du Québec en Abitibi-Témiscamingue (UQAT), Rouyn-Noranda, QC, Canada

**Keywords:** Opioids, Doctor shopping, Chronic noncancer pain, Overdose, Problematic opioid use, Opioid use disorder

## Abstract

**Introduction:**

Prescription opioids continue to be involved in the opioid crisis, and a better understanding of factors associated with problematic opioid use is needed.

**Objectives:**

The aim of this study was to assess the incidence of opioid doctor shopping, a proxy for problematic opioid use, to identify associated risk factors, and to assess its association with the occurrence of opioid overdoses.

**Methods:**

This was a retrospective cohort study of people living with chronic noncancer pain (CNCP) and treated with opioids for at least 6 months between 2006 and 2017 in the province of Quebec (Canada). Data were drawn from the Quebec health administrative databases. Doctor shopping was defined as overlapping prescriptions written by ≥ 2 prescribers and filled in ≥3 pharmacies.

**Results:**

A total of 8,398 persons with CNCP were included. The median age was 68.0 (Q1: 54; Q3: 82) years, and 37.1% were male. The 1-year incidence of opioid doctor shopping was 7.8%, 95% confidence interval (CI): 7.2–8.5. Doctor shopping was associated with younger age (hazard ratio [HR] 18–44 vs ≥65 years: 2.22, 95% CI: 1.77–2.79; HR 45–64 vs ≥65 years: 1.34, 95% CI: 1.11–1.63), male sex (HR = 1.20, 95% CI: 1.01–1.43), history of substance use disorder (HR = 1.32, 95% CI: 1.01–1.72), and anxiety (HR = 1.41, 95% CI: 1.13–1.77). People who exhibited doctor shopping were 5 times more likely to experience opioid overdoses (HR = 5.25, 95% CI: 1.44–19.13).

**Conclusion:**

Opioid doctor shopping is a marginal phenomenon among people with CNCP, but which is associated with the occurrence of opioid overdoses. Better monitoring of persons at high risk to develop doctor shopping could help prevent opioid overdoses.

## Abstract 2

Opioid doctor shopping was a rare phenomenon among people living with chronic noncancer pain but was associated with the occurrence of opioid overdoses.

## 1. Introduction

The opioid overdose crisis in the United States and in Canada has led to a high rate of opioid-related hospitalizations and overdoses and has become a public health concern.^35,44^ In 2019, an average of 38 people died each day in the United States from overdoses involving prescription opioids, totalling more than 14,000 deaths.^40^ In Canada, 19,355 opioid-related deaths occurred between January 2006 and September 2020.^45^ Although most of these opioid-related deaths involved illicitly manufactured fentanyl,^9,45^ a significant proportion of these deaths was related to prescription opioids.^28,30^ In the United States, prescription opioids were involved in 28% of all opioid overdose deaths in 2019, whereas in Canada, they were involved in 21% of opioid-related deaths in 2020.^45^ To address this crisis, it is important to know the extent of problematic opioid use (ie, using prescribed opioids in a manner not intended or instructed by a doctor or a pharmacist^26^) and to better monitor high-risk persons to prevent opioid-related deaths.

Doctor shopping, which consists of consultations with multiple physicians and/or pharmacies to obtain overlapping prescriptions, has been proposed as a relevant proxy for problematic opioid use.^5,42^ Indeed, doctor shopping was shown to be associated with opioid use disorder.^12,20^ Such a practice also disrupts continuity of care,^5^ does not allow adequate monitoring of the benefits and risks associated with opioid treatment, and exposes people to serious drug interactions. Furthermore, there is perhaps an association between doctor shopping and the occurrence of opioid overdoses^24,25,38^ but further studies are needed to establish this link. Thus, detection and monitoring opioid doctor shopping could help reduce inappropriate access to opioids and prevent opioid overdoses. In addition, early detection of risky behaviours involving prescription opioids such as opioid doctor shopping can assist prescribers in implementing safer prescribing practices. Although Canada is one of the countries where the opioid crisis is raging, no studies have been conducted on the occurrence of opioid doctor shopping among people living with chronic noncancer pain (CNCP). Thus, this study aims to better document opioid doctor shopping and its correlates by estimating its 1-year incidence among people with CNCP, identifying the risk factors associated with such behaviours, and assessing the relationship between doctor shopping and opioid overdoses.

## 2. Methods

### 2.1. Study design

This was a retrospective cohort study of people living with CNCP who lived in the province of Quebec (Canada) and were treated with opioids. Data from the Quebec health administrative databases were used to conduct this study.

### 2.2. Data sources

Data were drawn from the Quebec health insurance claims databases (*Régie de l'assurance maladie du Québec* [RAMQ]) and databases from the Quebec Ministry of Health and Social Services (*Ministère de la Santé et des Services sociaux* [MSSS]). Access to these databases was made possible through a tripartite agreement between the MSSS, the RAMQ, and the *Institut national d'excellence en santé et en services sociaux* (INESSS). These databases contain information from reimbursed services dispensed to people covered by the Quebec health insurance. A common and unique identifier for each recipient allowed to match information from these databases. The Quebec health insurance covers all Quebec residents for medical, hospital, and emergency services, and approximately 46% of Quebec residents for prescription drugs. The population who benefit from the prescription drug plan comprises persons aged 65 years and older, recipients of social assistance as well as workers who are not covered by a private drug insurance plan.

Ethical approval for this study was obtained from the Research Ethics Board of the *Centre hospitalier de l’Université de Montréal* and from the Quebec Research Ethics Board (*Commission d'accès à l'information du Québec*).

### 2.3. Participants

Persons aged 18 years and older and treated continuously with opioids for at least 6 consecutive months (183 days) between 2006 and 2017 were identified as living with chronic pain and on long-term opioid therapy. This selection strategy was based on the definition of chronic pain—ie, pain lasting for more than 3 months.^48^ A previous study showed that 3 months of continuous opioid use was associated with high specificity for identifying chronic pain patients^47^ but to be more conservative, we used 6 months of continuous opioid use. This selection method represented an alternative to the use of chronic pain diagnosis codes, which are underreported in the Quebec health insurance databases.^33^ In addition, this selection strategy has been used in 2 recent studies on opioid doctor shopping^13,14^ and allowed a comparison of our results with these previous studies. A continuous treatment was defined as an interval of 7 days or less between the end and the start of 2 consecutive opioid dispensations. The index date was the calendar date of the first opioid dispensation of the continuous treatment for at least 6 months. People with 5-year past International Statistical Classification of Diseases ninth revision (ICD-9) or 10th revision (ICD-10) diagnosis of cancer were excluded, and the remaining ones were identified as people living with CNCP. People with opioid use in the 6 months preceding the index date and less than 12 months of follow-up after the index date were excluded; people living with CNCP starting long-term opioid therapy with at least 12 months of follow-up comprised the final sample.

### 2.4. Procedures

#### 2.4.1. Demographic characteristics

Demographic characteristics included age, sex, and date of death as well as the eligibility for the health insurance plan and the drug insurance plan.

#### 2.4.2. Pharmaceutical services

Information on prescribed drugs included data such as the date of the dispensation, international nonproprietary name (INN) with the corresponding code, dose, and duration of treatment. Anonymous unique identifier and specialty of the drug prescriber along with the anonymous unique identifier of the pharmacy where the drug was dispensed were also recorded. Drugs were identified by using the INN codes. Opioids comprised codeine (including combination with acetaminophen), dextropropoxyphene (withdrawn from the market since 2010), fentanyl, hydromorphone, hydrocodone (except combination with phenylephrine or phenyltoloxamine commonly prescribed to treat coughs), meperidine, morphine, oxycodone (including combination with acetaminophen, acetylsalicylic acid or naloxone), tapentadol, tramadol (including combination with acetaminophen), butorphanol, and pentazocine. To include only people who use opioids to relieve pain, methadone and buprenorphine were excluded because they are commonly used as opioid agonists to treat opioid use disorders. Coprescription drugs were also collected and classified as benzodiazepines, antidepressants, antipsychotics, mood stabilizers, antiepileptics, central nervous system stimulants, and muscle relaxants. Coprescription drugs were classified according to the Anatomical Therapeutic Chemical Classification System of the World Health Organization.

Previous drug use was defined as at least one drug dispensation in the 3 months preceding the index date, whereas coprescription drug use was defined as at least one drug dispensation between the index date and the end of the follow-up.

#### 2.4.3. Medical visits, emergency, and hospital services

Medical services comprised the date of the visit to a physician, her/his medical specialty, and the ICD-9 diagnostic codes.

Information on emergency visits and hospitalizations included dates of admission and discharge, ICD-10 diagnosis codes at admission time, provenance for admission, and discharge destination. However, data on emergency visits are only available since 2012; thus, for comorbidity identification, only medical and hospitalization data were screened, whereas for emergency visits and hospitalizations for opioid overdoses, we included only people with an index date after 2013.

Identified comorbidities included substance use disorders (ICD-9 codes: 3030–3059; F10.0–F19.9), depression (ICD-9 codes: 2962, 2963, 2966–2968, 2980, 3004, 3090, 3091, 310–3119; ICD-10 codes: F30.0–F39.9), and anxiety disorders (ICD-9 codes: 3000–3003; ICD-10 codes: F064, F408 to F413, F418, F419, F931, F932). History of comorbidity was defined as at least one diagnosis code of the comorbidity in the past 12 months. For each comorbidity, medical services database and hospital services databases were screened to identify the corresponding ICD-9 and ICD-10 codes, respectively. Emergency visits and hospitalizations for opioid overdoses were identified through ICD-10 T400 to T406 and ICD-9 9650 diagnoses codes for reasons of admissions.

### 2.5. Outcomes

Opioid doctor shopping was defined as at least 1 day of overlapping prescriptions written by at least 2 different prescribers and filled in at least 3 different pharmacies. Each different overlapping prescription that met these criteria was considered as a new episode of doctor shopping. This definition was the same as the one used in the studies by Cepeda et al.^10,11^ and Chenaf et al.^13,14^ and has been shown to be associated with a diagnosis of opioid use disorder.^12^

### 2.6. Statistical analysis

#### 2.6.1. Participants' characteristics

Descriptive statistics (median, interquartile range [Q_1_–Q_3_]), and n (%) were used to portray the characteristics of the sample.

#### 2.6.2. Incidence of opioid doctor shopping

The one-year incidence of doctor shopping was estimated using the Kaplan-Meier method. The index date was the date of the first opioid prescription during the 12-month follow-up period, and the ending date was the date of the first episode of opioid doctor shopping (or of last information—ie, death, end of opioid treatment, switch to another analgesic, or end of follow-up). Time to the first episode of doctor shopping and number of episodes during the follow-up period were also computed. Comparisons between people who exhibited opioid doctor shopping behaviours and those who did not were conducted using χ^2^ test for frequencies ≥5 and Fisher exact test for frequencies <5.

#### 2.6.3. Risk factors of opioid doctor shopping

Cox proportional hazards models were applied to identify factors associated with opioid doctor shopping. Relevant variables to be included in the analysis were selected based on the existing scientific literature and their clinical relevance. The proportional hazard assumption was tested using the scaled Schoenfeld residuals. Assumption is met if the sum of Schoenfeld residuals was equal or very close to zero. Only variables that achieved this assumption were included in the analysis. Univariable analysis was performed to study the relation between each independent variable and the dependent variable (doctor shopping). Multivariable analysis was then conducted to study the association between each factor and doctor shopping, adjusting for confounders. Hazard ratios and their 95% confidence intervals were reported. The level of statistical significance was fixed at 0.05.

#### 2.6.4. Association between opioid doctor shopping and opioid overdoses

To assess the association between opioid doctor shopping and opioid overdoses, Marginal Structural Cox Models (Cox-MSM) were applied. Considering that emergency visit data were only available since 2012, we included only people with an index date beginning in 2013 to allow one year to screen for previous opioid overdose. People with past-year opioid overdoses were excluded because it was a strong predictor of occurrence of new episodes of overdoses. The follow-up was split into 4 time points of 3 months each. Doctor shopping, overdose, coprescriptions, and comorbidities were recorded in each time interval. The ending period time was the time point when the first episode of overdose occurred.

At each time point, logistic regression was used to estimate the probability of developing doctor shopping based on previous values of the covariates (coprescriptions, comorbidities, and sociodemographics), including potential time-varying confounders. The inverse of these probabilities was generated to obtain the inverse-probability-of-treatment weights (IPTW). At each time point, logistic regression was also used to estimate the probability of developing opioid doctor shopping considering a previous episode of doctor shopping. This probability was used to multiply the IPTW generated previously to obtain stabilized weights by reducing their variability. Considering all participants completed a 12-month follow-up, and none was lost in follow-up (no censoring), the censoring weights were not estimated. Thus, only the stabilized IPTW generated previously were used to adjust the final Cox-MSM modelling the effect of doctor shopping on the occurrence of opioid overdoses. Hazard ratios and their 95% confidence intervals were reported. The level of statistical significance was fixed at 0.05. All analyses were conducted with Stata 15.1 (StataCorp LLC) for Windows.

## 3. Results

### 3.1. Participants' inclusion

#### 3.1.1. Participants' characteristics

**Figure 1.**
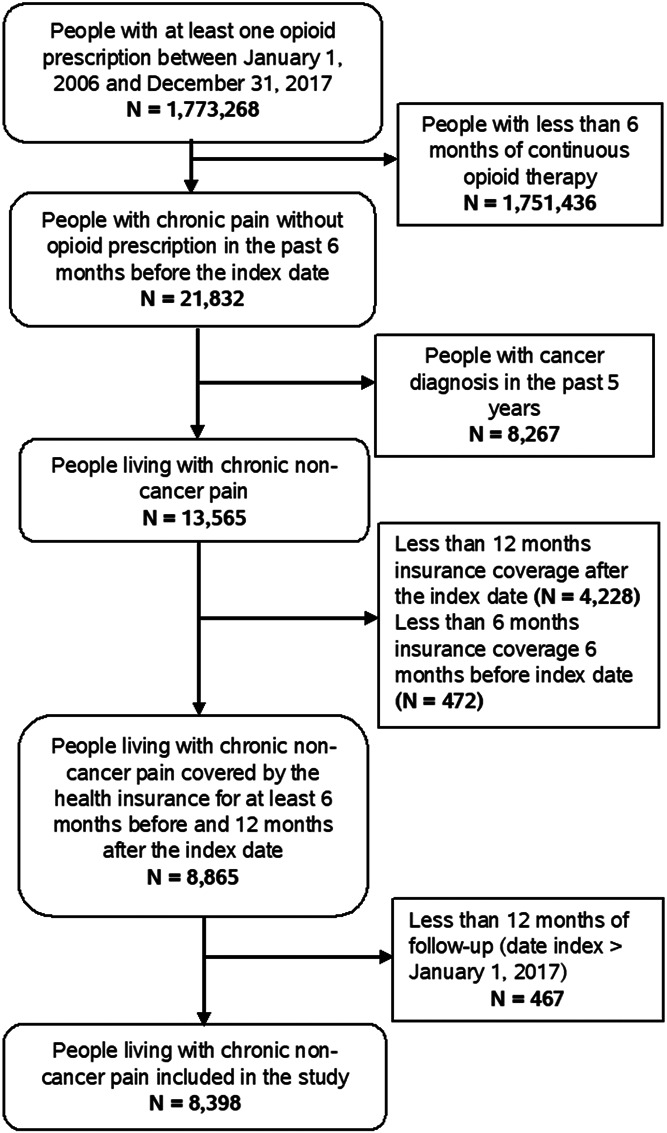
Flowchart of participants' inclusion.

A total of 8,398 persons were eligible between 2006 and 2017 (Fig. 1). Their median age was 68 years (Q_1_ = 54; Q_3_ = 82), and 37.1% were male. The percentage of people presenting a diagnosis of anxiety disorder was 13.4%, whereas 11.6% had a diagnosis of depression and 7.1% a diagnosis of substance use disorder. Among the included participants, 44.0% had used benzodiazepines and 38.7% had used antidepressants in the past 3 months (Table 1).

**Table 1:** Characteristics of participants included in the analysis identifying risk factors of opioid doctor shopping.

Variable	All	Doctor shopping		*P*
		No	Yes	
	n (%)	n (%)	n (%)	
N	8398 (100)	7789 (92.8)	609 (7.2)	
Sociodemographics				
Age				
Median (Q1–Q3)	68 (54–82)	69 (55–82)	60 (47–75)	<0.001
18 ≤ age < 45 y	919 (10.9)	792 (10.2)	127 (20.9)	<0.001
45 ≤ age < 65 y	2 687 (32.0)	2 473 (31.7)	214 (35.1)	
Age ≥ 65 y	4 792 (57.1)	4 524 (58.1)	268 (44.0)	
Males	3 117 (37.1)	2 836 (36.4)	281 (46.1)	<0.001
Comorbidities in the past year				
Substance use disorder	595 (7.1)	527 (6.8)	68 (11.2)	<0.001
Depression disorder	975 (11.6)	899 (11.5)	76 (12.5)	0.487
Anxiety disorder	1 126 (13.4)	1 024 (13.2)	102 (16.8)	0.012
Coprescription drugs in the past 3 mo				
Benzodiazepines	3 696 (44.0)	3 472 (44.6)	224 (36.8)	<0.001
Antidepressants	3 252 (38.7)	3 058 (39.3)	194 (31.9)	<0.001
Antipsychotics	1 411 (16.8)	1 330 (17.1)	81 (13.3)	0.016
Mood stabilizers	68 (0.8)	60 (0.8)	8 (1.3)	0.150
Antiepileptics	2 244 (26.7)	2 112 (27.1)	132 (21.7)	0.003
Central nervous system stimulants	63 (0.8)	59 (0.8)	4 (0.7)	0.782
Muscle relaxants	639 (7.6)	582 (7.5)	57 (9.4)	0.091

### 3.2. One-year incidence of opioid doctor shopping

Among the 8,398 participants included, 609 (7.2%) presented at least one episode of opioid doctor shopping during the 12-month follow-up after the index date. The median time elapsed between the first opioid dispensation (index date) and the first episode of doctor shopping was 88 days (Q_1_ = 39; Q_3_ = 166). The one-year cumulative incidence of doctor shopping was 7.8% (95% CI: 7.2–8.5).

Among opioid doctor shoppers, only one episode was recorded for 337 of them (55.3%), 2 episodes for 115 (18.9%), 3 episodes for 58 (9.5%), 4 episodes for 44 (7.2%), 5 episodes for 20 (3.3%), whereas 35 (5.7%) participants presented 6 episodes or more. The maximum number of episodes was 24 and was exhibited by only one person.

Table 1 compares participants who exhibited at least one episode of opioid doctor shopping behaviours and those who did not. The former group was slightly but significantly younger on the average and included a greater proportion of males. People who exhibited doctor shopping were also more likely to have a history of substance use disorders and anxiety disorders.

### 3.3. Risk factors of opioid doctor shopping

The results of the multivariable analysis revealed that opioid doctor shopping was significantly associated with younger age (HR = 2.22, 95% CI:1.77–2.79) for 18 ≤ age <45 years and HR = 1.34, 95% CI: 1.11 to 1.63 for 45 ≤ age<65 years vs ≥65 years and male sex (HR = 1.20, 95% CI: 1.20–1.43). Participants with a history of substance use disorder or anxiety disorder were also at higher risk to exhibit doctor shopping with HR = 1.32, 95% CI: 1.01 to 1.72 and HR = 1.41, 95% CI: 1.13 to 1.77, respectively. The use of mood stabilizers was also associated with doctor shopping (HR = 2.10, 95% CI: 1.03–4.27). By contrast, use of benzodiazepines (HR = 0.83, 95% CI: 0.70–0.99), antidepressants (HR = 0.77, 95% CI: 0.64–0.93), antipsychotics (HR = 0.72, 95% CI: 0.56–0.93), and antiepileptics (HR = 0.80, 95% CI: 0.66–0.98) in the past 3 months was negatively associated with the occurrence of opioid doctor shopping behaviours (Table 2).

**Table 2:** Cox proportional hazards univariable and multivariable analyses identifying risk factors associated with opioid doctor shopping.

Factors	Cox proportional hazards univariable analysis		Cox proportional hazards multivariable analysis	
	HR (95% CI)	*P*	HR (95% CI)	*P*
Sociodemographics				
Age				
≥ 65 y	Ref.		Ref.	
45 ≤ age < 65 y	1.44 (1.20–1.72)	<0.001	1.34 (1.11–1.63)	0.003
18 ≤ age < 45 y	2.57 (2.08–3.18)	<0.001	2.22 (1.77–2.79)	<0.001
Sex				
Female	Ref.		Ref.	
Male	1.47 (1.26–1.73)	<0.001	1.20 (1.01–1.43)	0.034
Past year comorbidities				
Substance use disorder				
No	Ref.		Ref.	
Yes	1.67 (1.30–2.15)	<0.001	1.32 (1.01–1.72)	0.044
Depression disorder				
No	Ref.			
Yes	1.09 (0.86–1.38)	0.488	1.03 (0.79–1.33)	0.834
Anxiety disorder				
No	Ref.		Ref.	
Yes	1.31 (1.06–1.62)	0.012	1.41 (1.13–1.77)	0.003
Coprescription drugs used in the past 3 mo				
Benzodiazepines				
No	Ref.		Ref.	
Yes	0.72 (0.61–0.85)	<0.001	0.83 (0.70–0.99)	0.045
Antidepressants				
No	Ref.		Ref.	
Yes	0.72 (0.61–0.86)	<0.001	0.77 (0.64–0.93)	0.007
Antipsychotics				
No	Ref.		Ref.	
Yes	0.74 (0.59–0.94)	0.013	0.72 (0.56–0.93)	0.011
Mood stabilizers				
No	Ref.		Ref.	
Yes	1.67 (0.83–3.35)	0.151	2.10 (1.03–4.27)	0.042
Antiepileptics				
No	Ref.		Ref.	
Yes	0.74 (0.61–0.90)	0.002	0.80 (0.66–0.98)	0.030
Central nervous system stimulants				
No	Ref.		Ref.	
Yes	0.88 (0.33–2.35)	0.795	0.76 (0.28–2.03)	0.580
Muscle relaxants				
No	Ref.		Ref.	
Yes	1.26 (0.96–1.65)	0.100	1.16 (0.88–1.54)	0.288

HR (95% CI), hazard ratio (95% confidence interval).

### 3.4. Association between opioid doctor shopping and opioid overdoses

Among the 8,398 participants included, 4,945 were excluded from this subanalysis because the index date was anterior to 2013. In addition, 16 persons were excluded because of history of opioid overdoses in the past 12 months. Thus, 3,437 persons were included in this subanalysis and among them, 25 (0.73%) experienced opioid overdoses. The characteristics of persons included in this subanalysis are presented in Table 3.

**Table 3:** Characteristics of participants included in the analysis estimating the association between opioid doctor shopping and the occurrence of opioid overdoses.

Variable	All	Opioid overdose		*P*
		No	Yes	
	n (%)	n (%)	n (%)	
N	3437	3412 (99.3)	25 (0.7)	
Opioid doctor shopping	243 (7.1)	239 (7.0)	4 (16.0)	
Sociodemographics				
Age				
Median (Q1–Q3)	69 (55–83)	69 (56–83)	52 (44–62)	<0.001
18 ≤ age < 45 y	345 (10.0)	338 (9.9)	7 (28.0)	<0.001
45 ≤ age < 65 y	1 082 (31.5)	1 069 (31.3)	13 (52.0)	
age ≥ 65 y	2 010 (58.5)	2 005 (58.8)	5 (20.0)	
Male	1 324 (38.5)	1 313 (38.5)	11 (44.0)	0.572
Comorbidities in the past year				
Substance use disorder	238 (6.9)	233 (6.8)	5 (20.0)	0.010
Depression disorder	378 (11.0)	370 (10.8)	8 (32.0)	0.001
Anxiety disorder	445 (13.0)	438 (12.8)	7 (28.0)	0.024
Coprescription drugs used in the past 3 mo				
Benzodiazepines	1 425 (41.5)	1 410 (41.3)	15 (60.0)	0.059
Antidepressants	1 370 (39.9)	1 357 (39.8)	13 (52.0)	0.213
Antipsychotics	657 (19.1)	649 (19.0)	8 (32.0)	0.100
Mood stabilizers	24 (0.7)	23 (0.7)	1 (4.0)	0.047
Antiepileptics	1 249 (36.3)	1 239 (36.3)	10 (40.0)	0.703
Central nervous system stimulants	29 (0.8)	28 (0.8)	1 (4.0)	0.083
Muscle relaxants	279 (8.1)	273 (8.0)	6 (24.0)	0.004

In the Cox-MSM without adjustment, doctor shopping was linked to the occurrence of opioid overdose with HR = 8.48, 95% CI: 2.47–29.13, *P* = 0.001. In the final model using stabilized IPTW for adjustment, doctor shopping remained significantly linked to opioid overdoses with HR = 5.25, 95% CI: 1.44–19.13, *P* = 0.012.

## 4. Discussion

This study is the first of its kind to assess opioid doctor shopping among people living with CNCP in the province of Quebec (Canada). The study highlights that only a minority of people living with CNCP engages in doctor shopping, identifies the associated factors of this behaviour, and establishes a link between opioid doctor shopping and the occurrence of opioid overdoses.

Doctor shopping refers to many behaviours and can be practised for different reasons other than nonmedical use such as for convenience, prescriber and drug unavailability, or price.^42,46^ However, the conservative definition used in this study encompassed prescription overlapping with multiple prescribers and pharmacies and thereby looked at intentional behaviours to get large quantities of opioids for nonmedical use. Documenting the incidence of opioid doctor shopping is useful for clinicians to better monitor persons at high risk of problematic opioid use and informative for health care decision makers to implement appropriate measures regarding the extent of this behaviour and its consequences.

### 4.1. Rate of opioid doctor shopping

In this study, the one-year incidence of opioid doctor shopping was lower than 8% but more than half of the shoppers (55.5%) exhibited only one episode. This incidence rate is higher than the ones reported in the studies conducted by Cepeda et al. in the United States before the worsening of opioid crisis (0.18% to 0.30%).^10,11^ This surprising result, given that the United States is the country most affected by the opioid crisis, could be due to methodological differences. Indeed, Cepeda et al.^10,11^ included all persons who had at least one opioid prescription, which has the effect of increasing the denominator and thereby decreasing the incidence rate. Two previous studies conducted in France and using the same methodology as the one we used—ie, same definition of doctor shopping and CNCP—also reported lower incidence rates of opioid doctor shopping of 1 to 4%^13,14^ compared to the one observed in this study. This could be the differences in opioid prescribing practices. Indeed, weak opioids were the most prescribed opioids in France, whereas in Quebec, strong opioids that have a higher potential for nonmedical use were the most prescribed.^15^ However, these incidence rates are not alarming and suggest that only a minority of people living with CNCP engage in opioid doctor shopping behaviours. Such findings conflict with the prejudice and stigma towards people living with CNCP, who are sometimes seen as people dependent to their medications or as drug-seekers.^18,31^ Nevertheless, best practices must be promoted to prevent opioid doctor shopping and improve opioid prescribing by screening for risk factors of developing this type of behaviour before and during prescribing opioids.

### 4.2. Factors associated with opioid doctor shopping

Several risk factors associated with opioid doctor shopping were identified in this study. Younger people and men were at higher risk to engage in this type of behaviour, a finding which is consistent with previous studies.^5,16,17,27^ Data on the opioid crisis in Canada revealed that among opioid-related deaths, 67% occurred in people younger than 50 years of age and 75% involved men.^45^ The association between young age and opioid use disorders is also well documented.^17,49^ Two studies have shown that the most common motives for the nonmedical use of opioids among young people were to get high and to experiment.^36,37^ Factors such as stress and anxiety often present in young people may also conduct to nonmedical use of opioids to cope with these states.^1,29^ The sex difference in opioid use disorders would be the result of biological and sociocultural differences.^2,3,21^ For example, Fattore et al. have argued that the sense of responsibility and fear of addiction stigma could protect women from developing opioid use disorders and behaviours such as doctor shopping.^21^ By contrast, men would be more susceptible to develop problematic opioid use due to impulsivity, peer pressure, and the need of belonging to a group.^21,39^

The results of our study showed that history of substance use disorder was associated with a higher risk of opioid doctor shopping. This is consistent with the fact that past substance use disorder is known to be a strong predictor of opioid use disorder.^7^ We also found that history of anxiety disorder was a significant predictor of opioid doctor shopping. Some studies also found a significant association between past anxiety disorder and problematic substance use.^22,33,41^

Among other risk factors of opioid doctor shopping, we found that past use of psychotropic drugs such as antidepressants, antipsychotics, and benzodiazepines was associated with a lower incidence of such a type of behaviour. This striking finding could suggest that these medications allow people to manage well health conditions that normally increase the risk of problematic opioid use. Indeed, among reasons that have been shown to lead to opioid use disorders, self-medication of undertreated pain, depression, anxiety, and sleep problems were commonly cited.^36,43,49^ However, further studies are needed to better understand this type of association and to assess whether the risk of developing problematic opioid use is associated with underlying mental disorders and/or psychotropic medication. Despite the evidence that some factors were associated with risk of developing opioid doctor shopping, it remains that opioids are essential to relieve CNCP in some persons and concerns about the development of opioid use disorder should not prevent proper pain management. Effective communication between physicians and patients along with frequent reevaluations of the benefit/risk ratio of opioid therapy can help improve the adequacy of long-term opioid therapy and reduce the incidence of doctor shopping.

### 4.3. Opioid doctor shopping and risk of overdose

Another important finding of this study is that opioid doctor shopping increased the risk of opioid overdoses. People who exhibited doctor shopping were 5 times more likely to experience opioid overdoses, although confidence intervals were large due to the low number of overdoses in our sample. Some previous studies also reported that visiting multiple prescribers and pharmacies to obtain opioids predicted opioid overdoses and deaths.^24,25,38^ These findings help understanding consequences of opioid doctor shopping and suggest doctor shoppers use drugs for themselves, thus increasing the risk of opioid-related overdoses. The implementation of effective opioid prescription monitoring program could help reduce doctor shopping and the associated overdoses in people who access opioids through medical providers.^8^ However, the implementation of prescription monitoring program to reduce access to prescription opioids for nonmedical use could lead to an increase in use of illicitly manufactured fentanyl or heroin.^6,8,19^ Furthermore, most of the opioid overdoses did not involve doctor shopping and the opioid overdose crisis was driven mainly by illicit fentanyl. Thus, complementary measures other than preventing opioid doctor shopping are needed to reduce the occurrence of overdoses. Better access to multidisciplinary pain management and nonpharmacological pain modalities may improve pain management, whereas better access to mental health services and opioid agonists may improve addiction management.

### 4.4. Study limitations and strengths

This study presents some limitations. As mentioned earlier, our sample did not include persons who had a private medication insurance plan. Some studies in Quebec^4^ and elsewhere in North America^23,34,50^ suggest that people benefitting from public medication insurance plan would have a lower socioeconomic status than those covered by a private plan. If this is the case, it may limit the generalizability of our findings but not the internal validity (capacity to detect valid associations). It is also important to consider that the rate of opioid doctor shopping could be different from one country to another or within a country, depending on the restrictions on changing doctors, the presence of a prescription monitoring system, or differences in medication accessibility and medication use patterns. In addition, confounding biases may have influenced the identification of risk factors. Indeed, it was not possible to include in our analysis factors such as pain characteristics (eg, intensity and duration, etc.) because such data were not available in the RAMQ databases. Further research is also needed to confirm the association we observed between opioid doctor shopping and opioid overdoses, considering the small sample size of people who exhibited doctor shopping.

Despite its limitations, this study provides useful information on opioid doctor shopping and its correlates. Several relevant factors were identified, allowing better screening of persons at high risk to develop such a type of behaviour. Furthermore, this study established a link between opioid doctor shopping and the occurrence of opioid overdoses, highlighting the serious risk that can be associated with this practice.

## 5. Conclusions

Opioid doctor shopping seems to be a marginal phenomenon among people with CNCP, but people who exhibited doctor shopping would be at higher risk of opioid overdoses. Younger age, male sex, history of anxiety, and substance use disorders were associated with higher risk of doctor shopping behaviours. The implementation of prescription monitoring systems may help reduce this phenomenon and prevent opioid overdoses. Furthermore, better access to multidisciplinary pain treatment and nonpharmacological pain modalities may help reduce and optimize opioid use, which subsequently could lead to a decrease in rates of opioid use disorders and overdoses.

## Disclosures

The authors have no conflicts of interest to declare.

## References

[1] Bandelow B Michaelis S. Epidemiology of anxiety disorders in the 21st century. Dialogues Clin Neurosci 2015;17:327–35. 26487813 10.31887/DCNS.2015.17.3/bbandelow PMC4610617

[2] Becker JB McClellan M Reed BG. Sociocultural context for sex differences in addiction. Addict Biol 2016;21:1052–9. 26935336 10.1111/adb.12383 PMC5555215

[3] Becker JB McClellan ML Reed BG. Sex differences, gender and addiction. J Neurosci Res 2017;95:136–47. 27870394 10.1002/jnr.23963 PMC5120656

[4] Bérard A Lacasse A. Validity of perinatal pharmacoepidemiologic studies using data from the RAMQ administrative database. Can J Clin Pharmacol 2009;16:e360–369. 19553702

[5] Biernikiewicz M Taieb V Toumi M. Characteristics of doctor-shoppers: a systematic literature review. J Market Access Health Pol 2019;7. doi: 10.1080/20016689.2019.1595953. PMC6442108 30956784

[6] Brown R Riley MR Ulrich L Kraly EP Jenkins P Krupa NL Gadomski A. Impact of New York prescription drug monitoring program, I-STOP, on statewide overdose morbidity. Drug Alcohol Depend 2017;178:348–54. 28692945 10.1016/j.drugalcdep.2017.05.023

[7] Busse JW Craigie S Juurlink DN Buckley DN Wang L Couban RJ Agoritsas T Akl EA Carrasco-Labra A Cooper L Cull C Costa BRda Frank JW Grant G Iorio A Persaud N Stern S Tugwell P Vandvik PO Guyatt GH. Guideline for opioid therapy and chronic noncancer pain. CMAJ 2017;189:E659–66. 28483845 10.1503/cmaj.170363 PMC5422149

[8] Castillo-Carniglia A Ponicki WR Gaidus A Gruenewald PJ Marshall BDL Fink DS Martins SS Rivera-Aguirre A Wintemute GJ Cerdá M. Prescription drug monitoring programs and opioid overdoses: exploring sources of heterogeneity. Epidemiology 2019;30:212–20. 30721165 10.1097/EDE.0000000000000950 PMC6437666

[9] Centers for Disease Control and Prevention. Annual surveillance report of drug-related risks and outcomes — United States surveillance special report. Centers for Disease Control and Prevention, U.S. Department of Health and Human Services, 2019. Available at: https://www.cdc.gov/drugoverdose/pdf/pubs/2019-cdc-drug-surveillancereport.pdf. Accessed April 20, 2021. n.d.:128.

[10] Cepeda MS Fife D Chow W Mastrogiovanni G Henderson SC. Assessing opioid shopping behaviour: a large cohort study from a medication dispensing database in the US. Drug Saf 2012;35:325–34. 22339505 10.2165/11596600-000000000-00000

[11] Cepeda MS Fife D Chow W Mastrogiovanni G Henderson SC. Opioid shopping behavior: how often, how soon, which drugs, and what payment method. J Clin Pharmacol 2013;53:112–17. 23400751 10.1177/0091270012436561

[12] Cepeda MS Fife D Kihm MA Mastrogiovanni G Yuan Y. Comparison of the risks of shopping behavior and opioid abuse between tapentadol and oxycodone and association of shopping behavior and opioid abuse. Clin J Pain 2014;30:1051–6. 24370606 10.1097/AJP.0000000000000067 PMC4232297

[13] Chenaf C Kabore J-L Delorme J Pereira B Mulliez A Roche L Eschalier A Delage N Authier N. Codeine shopping behavior in a retrospective cohort of chronic noncancer pain patients: incidence and risk factors. J Pain 2016;17:1291–301. 27592608 10.1016/j.jpain.2016.08.010

[14] Chenaf C Kabore J-L Delorme J Pereira B Mulliez A Roche L Eschalier A Delage N Authier N. Incidence of tramadol shopping behavior in a retrospective cohort of chronic non-cancer pain patients in France. Pharmacoepidemiol Drug Saf 2016;25:1088–98. 27363310 10.1002/pds.4056

[15] Chenaf C Kaboré J-L Delorme J Pereira B Mulliez A Zenut M Delage N Ardid D Eschalier A Authier N. Prescription opioid analgesic use in France: trends and impact on morbidity-mortality. Eur J Pain 2019;23:124–34. 30051548 10.1002/ejp.1291

[16] Cochran BN Flentje A Heck NC Van Den Bos J Perlman D Torres J Valuck R Carter J. Factors predicting development of opioid use disorders among individuals who receive an initial opioid prescription: mathematical modeling using a database of commercially-insured individuals. Drug Alcohol Depend 2014;138:202–8. 24679839 10.1016/j.drugalcdep.2014.02.701 PMC4046908

[17] Cragg A Hau JP Woo SA Kitchen SA Liu C Doyle-Waters MM Hohl CM. Risk factors for misuse of prescribed opioids: a systematic review and meta-analysis. Ann Emerg Med 2019;74:634–46. 31229388 10.1016/j.annemergmed.2019.04.019

[18] Dassieu L Heino A Develay É Kaboré J-L Pagé MG Moor G Hudspith M Choinière M. “They think you’re trying to get the drug”: qualitative investigation of chronic pain patients' health care experiences during the opioid overdose epidemic in Canada. Can J Pain 2021;5:66–80. 34189391 10.1080/24740527.2021.1881886 PMC8210863

[19] Delcher C Wang Y Wagenaar AC Goldberger BA Cook RL Maldonado-Molina MM. Prescription and illicit opioid deaths and the prescription drug monitoring program in Florida. Am J Public Health 2016;106:e10–11. 10.2105/AJPH.2016.303104 PMC4880260 27153025

[20] Esposito DB Cepeda MS Lyons JG Yin R Lanes S, O behalf of TM of the OP-MCOSW Group. Medical record-based ascertainment of behaviors suggestive of opioid misuse, diversion, abuse, and/or addiction among individuals showing evidence of doctor/pharmacy shopping. J Pain Res 2019;12:2291. 31413626 10.2147/JPR.S203350 PMC6661981

[21] Fattore L Melis M Fadda P Fratta W. Sex differences in addictive disorders. Front Neuroendocrinol 2014;35:272–84. 24769267 10.1016/j.yfrne.2014.04.003

[22] Fridell M Bäckström M Hesse M Krantz P Perrin S Nyhlén A. Prediction of psychiatric comorbidity on premature death in a cohort of patients with substance use disorders: a 42-year follow-up. BMC Psychiatry 2019;19:150. 31092225 10.1186/s12888-019-2098-3 PMC6518448

[23] Fronstin P. Sources of health insurance and characteristics of the uninsured: analysis of the March 2007 Current Population Survey. EBRI Issue Brief 2007:1–33. 17987754

[24] Gwira Baumblatt JA Wiedeman C Dunn JR Schaffner W Paulozzi LJ Jones TF. High-risk use by patients prescribed opioids for pain and its role in overdose deaths. JAMA Intern Med 2014;174:796–801. 24589873 10.1001/jamainternmed.2013.12711

[25] Hall AJ Logan JE Toblin RL Kaplan JA Kraner JC Bixler D Crosby AE Paulozzi LJ. Patterns of abuse among unintentional pharmaceutical overdose fatalities. JAMA 2008;300:2613–20. 19066381 10.1001/jama.2008.802

[26] Health Canada. Opioids and the opioid crisis – get the facts, 2018. Available: https://www.canada.ca/en/health-canada/services/substance-use/problematic-prescription-drug-use/opioids/get-the-facts.html#a5. Accessed 27 Apr 2021.

[27] Hudgins JD Porter JJ Monuteaux MC Bourgeois FT. Prescription opioid use and misuse among adolescents and young adults in the United States: a national survey study. PLOS Med 2019;16:e1002922. 31689290 10.1371/journal.pmed.1002922 PMC6830740

[28] Jones MR Viswanath O Peck J Kaye AD Gill JS Simopoulos TT. A brief history of the opioid epidemic and strategies for pain medicine. Pain Ther 2018;7:13–21. 29691801 10.1007/s40122-018-0097-6 PMC5993682

[29] Kessler RC Petukhova M Sampson NA Zaslavsky AM Wittchen H-U. Twelve-month and lifetime prevalence and lifetime morbid risk of anxiety and mood disorders in the United States. Int J Methods Psychiatr Res 2012;21:169–84. 22865617 10.1002/mpr.1359 PMC4005415

[30] Kolodny A Courtwright DT Hwang CS Kreiner P Eadie JL Clark TW Alexander GC. The prescription opioid and heroin crisis: a public health approach to an epidemic of addiction. Annu Rev Public Health 2015;36:559–74. 25581144 10.1146/annurev-publhealth-031914-122957

[31] Lacasse A Choinière M Connelly J-A. Knowledge, beliefs, and attitudes of the Quebec population toward chronic pain: where are we now?. Can J Pain 2017;1:151–60. 35005351 10.1080/24740527.2017.1369849 PMC8730576

[32] Lacasse A Ware MA Dorais M Lanctôt H Choinière M. Is the Quebec provincial administrative database a valid source for research on chronic non-cancer pain? Pharmacoepidemiol Drug Saf 2015;24:980–90. 26105572 10.1002/pds.3820

[33] Lai HMX Cleary M Sitharthan T Hunt GE. Prevalence of comorbid substance use, anxiety and mood disorders in epidemiological surveys, 1990-2014: a systematic review and meta-analysis. Drug Alcohol Depend 2015;154:1–13. 26072219 10.1016/j.drugalcdep.2015.05.031

[34] Lesser IM Leuchter AF Trivedi MH Davis LL Wisniewski SR Balasubramani GK Petersen T Stegman D Rush AJ. Characteristics of insured and noninsured outpatients with depression in STAR(*)D. Psychiatr Serv 2005;56:995–1004. 16088018 10.1176/appi.ps.56.8.995

[35] Lisa B Jessica H. Evidence synthesis - the opioid crisis in Canada: a national perspective. Health Promot Chronic Dis Prev Can 2018;38:224–33. 29911818 10.24095/hpcdp.38.6.02 PMC6034966

[36] Lord S Brevard J Budman S. Connecting to young adults: an online social network survey of beliefs and attitudes associated with prescription opioid misuse among college students. Subst Use Misuse 2011;46:66–76. 21190407 10.3109/10826084.2011.521371 PMC4201950

[37] McCabe SE West BT Boyd CJ. Motives for medical misuse of prescription opioids among adolescents. The J Pain 2013;14:1208–16. 23954519 10.1016/j.jpain.2013.05.004 PMC3792708

[38] Peirce GL Smith MJ Abate MA Halverson J. Doctor and pharmacy shopping for controlled substances. Med Care 2012;50:494–500. 22410408 10.1097/MLR.0b013e31824ebd81

[39] Peters L Soyka M. Interrelationship of opioid dependence, impaired impulse control, and depressive symptoms: an open-label cross-sectional study of patients in maintenance therapy. Neuropsychobiology 2019;77:73–82. 30453290 10.1159/000494697

[40] Prescription opioid overdose death maps | drug overdose | CDC injury center. 2021. Available: https://www.cdc.gov/drugoverdose/data/prescribing/overdose-death-maps.html. Accessed 19 Apr 2021.

[41] Rogers AH Kauffman BY Bakhshaie J McHugh RK Ditre JW Zvolensky MJ. Anxiety sensitivity and opioid misuse among opioid-using adults with chronic pain. Am J Drug Alcohol Abuse 2019;45:470–8. 30896985 10.1080/00952990.2019.1569670 PMC7137151

[42] Sansone RA Sansone LA. Doctor shopping: a phenomenon of many themes. Innov Clin Neurosci 2012;9:42–6. PMC3552465 23346518

[43] Savage SR. What to do when pain and addiction coexist. J Fam Pract 2013;62:S10–16. 23828808

[44] Scholl L. Drug and opioid-involved overdose deaths—United States, 2013–2017. MMWR Morb Mortal Wkly Rep 2019;67:1419–1427. 10.15585/mmwr.mm675152e1 PMC6334822 30605448

[45] Special advisory committee on the epidemic of opioid overdoses. Opioids and stimulant-related harms in Canada. Ottawa: Public Health Agency of Canada; 2021. Available at: https://health-infobase.canada.ca/substance-related-harms/opioids-stimulants. Accessed July 21, 2021. n.d.

[46] Stephenson JJ Cepeda MS Zhang J Dinh J Hall K Esposito DB Kern DM. The association between doctor and pharmacy shopping and self-reported misuse and abuse of prescription opioids: a survey study. J Pain Res 2020;13:689–701. 32308468 10.2147/JPR.S232409 PMC7140905

[47] Tian TY Zlateva I Anderson DR. Using electronic health records data to identify patients with chronic pain in a primary care setting. J Am Med Inform Assoc 2013;20:e275–280. 23904323 10.1136/amiajnl-2013-001856 PMC3861913

[48] Treede R-D Rief W Barke A Aziz Q Bennett MI Benoliel R Cohen M Evers S Finnerup NB First MB Giamberardino MA Kaasa S Kosek E Lavand’homme P Nicholas M Perrot S Scholz J Schug S, Smith BH, Svensson P, Vlaeyen JWS, Wang S-J. A classification of chronic pain for ICD-11. PAIN 2015;156:1003–7. 10.1097/j.pain.0000000000000160 PMC4450869 25844555

[49] Webster LR. Risk factors for opioid-use disorder and overdose. Anesth Analg 2017;125:1741–8. 29049118 10.1213/ANE.0000000000002496

[50] Wells KB Sherbourne CD Sturm R Young AS Burnam MA. Alcohol, drug abuse, and mental health care for uninsured and insured adults. Health Serv Res 2002;37:1055–66. 12236383 10.1034/j.1600-0560.2002.65.x PMC1464001

